# Selective bactericidal efficacy of 465-nm blue light phototherapy against standard and canine wound pathogens: An *in vitro* evaluation

**DOI:** 10.14202/vetworld.2025.2064-2071

**Published:** 2025-07-27

**Authors:** Pimsiri Ngowwatana, Naruepon Kampa, Somphong Hoisang, Suvaluk Seesupa, Duangdaow Khunbutsri, Saikam Chaimongkol, Preenun Jitasombuti, Supranee Jitpean, Thanikul Srithunyarat, Chalermkwan Nonthakotr, Nitaya Boonbal, Piyasak Wipoosak, Duangdaun Kaenkangploo

**Affiliations:** 1 Division of Surgery, Faculty of Veterinary Medicine, Khon Kaen University, Khon Kaen 40002, Thailand; 2 Veterinary Diagnostic Laboratory, Faculty of Veterinary Medicine, Khon Kaen University, Khon Kaen 40002, Thailand; 3 Veterinary Teaching Hospital, Faculty of Veterinary Medicine, Khon Kaen University, Khon Kaen, 40002, Thailand

**Keywords:** antimicrobial resistance, blue light therapy, *Escherichia coli*, photoinactivation, *Pseudomonas aeruginosa*, veterinary phototherapy

## Abstract

**Background and Aim:**

Antibiotic resistance poses a growing threat to wound management in veterinary medicine. Blue light phototherapy has emerged as a non-antibiotic bactericidal alternative with additional benefits for wound healing. However, its effectiveness in clinical veterinary contexts remains inadequately explored. This study evaluated the bactericidal efficacy of 465-nm blue light against standard pathogens and bacteria isolated from infected canine wounds, aiming to determine optimal energy doses for clinical use.

**Materials and Methods:**

Three standard bacterial strains – *Staphylococcus aureus* (American Type Culture Collection [ATCC] 25923), *Pseudomonas aeruginosa* (ATCC 27853), and *Escherichia coli* (ATCC 25922) – along with five clinical isolates from canine wounds, were exposed to 465-nm blue light at energy doses of 28, 56, and 112 J/cm^2^ (15, 30, and 60 min, respectively). Colony-forming units (CFUs) were quantified post-irradiation and compared to non-irradiated controls. Statistical significance was assessed using appropriate parametric and non-parametric tests.

**Results:**

*P. aeruginosa* (ATCC 27853) exhibited significant, dose-dependent inhibition at all energy doses, resulting in reductions of 36.3%, 60.5%, and 82.8%. Clinical *P. aeruginosa* isolates demonstrated 21.1% and 78.8% inhibition at 56 and 112 J/cm^2^, respectively (p < 0.05). *E. coli* (ATCC 25922) was significantly inhibited only at 112 J/cm^2^ (46.4% reduction, p = 0.045). No significant reductions were observed for *S. aureus*, *Acinetobacter baumannii*, *Staphylococcus haemolyticus*, clinical *E. coli*, or *Enterococcus faecalis* at any dose.

**Conclusion:**

Blue light at 465-nm exhibits selective bactericidal activity, effectively inhibiting *P. aeruginosa* and *E. coli* (ATCC), with efficacy dependent on bacterial species and applied energy dose. Its limited effect on other pathogens underscores the importance of species-specific treatment planning. Higher energy doses (112 J/cm^2^) may be required in unknown or mixed infections. Further investigation is recommended to refine device specifications and assess clinical utility in veterinary settings.

## INTRODUCTION

Antibiotic resistance is a growing global concern that demands immediate and effective countermea-sures. As such, alternative strategies for bacterial inactivation are necessary [1]. Among these, visible light, especially within the blue spectrum (400–470 nm) has demonstrated bactericidal properties without the need for exogenous photosensitizers. It has been demonstrated to be effective against bacteria [2–4], fungi [5], and viruses [6]. A key advantage of blue light lies in its ability to selectively target microbial cells while preserving host cell integrity [7, 8], making it a promising candidate for therapeutic applications. Unlike antibiotics, which act on specific cellular targets, blue light operates through a different mechanism [9], thereby reducing the likelihood of resistance development [10, 11], a feature that enhances its clinical appeal. Besides its antimicrobial action, blue light has also been used to support wound healing and treat dermatological conditions, such as psoriasis and skin ulcers, in humans [12, 13], as well as to enhance healing in rat models [14]. Its rapid effect, along with being cost-effective, easy to use, painless, and safe, positions blue light as a valuable tool for managing infected wounds [12, 15–17].

Blue light has demonstrated considerable antimicrobial effectiveness against various bacterial strains [2, 18–21]. For example, *in vitro* exposure to 470-nm blue light at 3 J/cm^2^ reduced methicillin-resistant *Staphylococcus aureus* (MRSA) by 30%, while increasing the dose to 55 J/cm^2^ achieved 90.4% inhibition [22]. In murine models, 415-nm blue light at doses of 41.4 and 108 J/cm^2^ completely eradicated MRSA infections both *in vitro* and *in vivo* within 30 min–24 h after the bacterial inoculation [23]. A separate study by Amin *et al*. [3] showed that a single 48 J/cm^2^ dose of blue light successfully suppressed *Pseudomonas aeruginosa* in mouse wound infections. Moreover, 465-nm blue light *in vitro* reduced MRSA colony counts by 93.3%–100% at energy levels of 56.25, 112.5, and 225 J/cm^2^, although it was ineffective against *Staphylococcus pseudintermedius* from canine skin samples [24]. Subse-quent investigation by Bae and Oh [25] has reported up to 95.7% inhibition of *S. pseudintermedius* using 465–470 nm blue light at comparable doses. Notably, the effectiveness of blue light varies across bacterial species and strains, necessitating tailored energy doses (J/cm^2^) for optimal inactivation.

Despite extensive evidence supporting the bactericidal potential of blue light in human medicine, its application in veterinary settings remains underexplored. Prior studies have demonstrated the efficacy of various blue light wavelengths, particularly those in the range of 400–470 nm, against a broad spectrum of pathogens, including *S. aureus*, *P. aeruginosa*, and *Escherichia coli*. However, inconsistencies in results, particularly among different bacterial strains and light parameters, highlight the need for species-specific investigations. Moreover, few studies have evaluated the efficacy of 465-nm blue light specifically on bacteria isolated from clinical canine wounds, which often harbor diverse and antibiotic-resistant organisms. There is also limited evidence regarding the optimal energy dose required for effective bacterial suppression under practical, clinically relevant conditions.

This study aimed to evaluate the bactericidal efficacy of 465-nm blue light phototherapy against both standard bacterial strains and clinical isolates obtained from infected canine wounds. Specifically, the study sought to determine whether 465-nm blue light could effectively reduce bacterial colony counts at varying energy doses (28, 56, and 112 J/cm^2^) and to identify dose-dependent responses among different bacterial species. The ultimate goal was to assess its feasibility as an adjunct or alternative to antibiotic therapy in veteri-nary wound management.

## MATERIALS AND METHODS

### Ethical approval

This study received ethical approval from the Institu-tional Animal Care and Use Committee of Khon Kaen University (Approval No. IACUC-KKU 50/67). Informed consent was obtained from the dog owners before the collection of wound samples for bacterial culture.

### Study period and location

This study was conducted from June 2024 to January 2025 at the Faculty of Veterinary Medicine, Khon Kaen University, Khon Kaen, Thailand.

### Sample size determination

The number of experimental replicates (n) was calculated using the method for comparing two independent means. The effect size was estimated from preliminary data obtained in a pilot study. A significance level of 0.05 and a statistical power of 0.8 were applied. Based on these parameters, the required replication was determined to be quadruplicate (n = 4).

### Bacterial strains and clinical isolates

This study utilized three standard bacterial strains: *S. aureus* (American Type Culture Collection [ATCC] 25923), *P. aeruginosa* (ATCC 27853), and *E. coli* (ATCC 25922). In addition, clinical bacterial isolates were obtained from infected canine wounds treated at the Veterinary Teaching Hospital, Faculty of Veterinary Medicine, Khon Kaen University, Thailand. Eligible wounds were <3 weeks old, exhibited discharge from any cause, and may have been exposed to antibiotics before sampling. The clinical strains used were selected based on common bacterial species identified in unpub-lished local surveillance data of canine wound infections.

### Bacterial isolation and preparation

Samples were collected using sterile cotton swabs and transported in Amies Transport medium (Citotest Labware, Jiangsu, China). Bacteria were cultured aerobically on sheep blood agar and MacConkey agar at 37°C for 24 h. Identification was performed using matrix-assisted laser desorption/ionization time-of-flight mass spectrometry (Bruker Daltonics GmbH, Bremen, Germany). Isolates were preserved in tryptic soy broth with 20% glycerol at −20°C. Before experimentation, frozen isolates were subcultured on sheep blood agar, and bacterial suspensions were standardized to 0.5 McFarland (~0.5 × 10^8^ colony-forming units [CFU]/mL) using a spectrophotometer (Epoch, BioTek, USA) at 600-nm. Serial 10-fold dilutions were performed to achieve approximately 10^4^ CFU/mL. An aliquot of 10 μL (yielding 20–200 CFUs) was spread onto 35-mm tryptic soy agar plates (HiMedia, India), matching the irradiation area. Control plates remained unexposed, while treatment plates were subjected to blue light irradiation. All plates were incubated simultaneously at 37°C for 24 h in ambient air, alongside positive and negative controls. Colony counts were performed manually.

### Blue light irradiation protocol

Irradiation was conducted using the MR4 ACTIVet Pro veterinary laser device (Multi Radiance Medical, Solon, OH, USA), which emits blue light at 465-nm (Table 1). Calibration was performed using a power meter (PM160 Si Sensor, Thorlabs, USA), which confirmed a power density of 31 mW/cm^2^ at a distance of 5 mm. Each uncovered plate was irradiated from a distance of 5 mm. Treatment groups received energy doses of 28, 56, and 112 J/cm^2^, corresponding to 15, 30, and 60 min of exposure, respectively. Each dose group was tested in quadruplicate, with corresponding unirradiated controls.

**Table 1:** Technical specifications of the blue-light irradiation device (MR4 ACTIVet PRO, Multi Radiance Medical, Solon, USA).

Visible blue light radiation	465-nm
Average blue light power	200 mW
Mode	Continuous
Radiation aperture	4 ± 0.4 cm^2^
Magnetic induction	35 ± 10 mT
Irradiation time	15, 30, 60 min
Energy density at the aperture	28, 56, and 112 J/cm^2^
Overall dimension	203 × 64 × 70 mm

### Statistical analysis

Colony count data were assessed for normality using the Shapiro–Wilk test. Results are expressed as mean ± standard deviation (SD). For each energy dose, comparisons between the irradiated and control groups were performed using a t-test (for normally distributed data) or the Mann–Whitney U test (for non-normally distributed data). Repeated measures across energy doses were analyzed using a one-way analysis of variance (ANOVA). A p < 0.05 was considered statistically significant. Analyses were conducted using STATA version 14.1 (StataCorp LLC, USA).

## RESULTS

**Figure 1:**
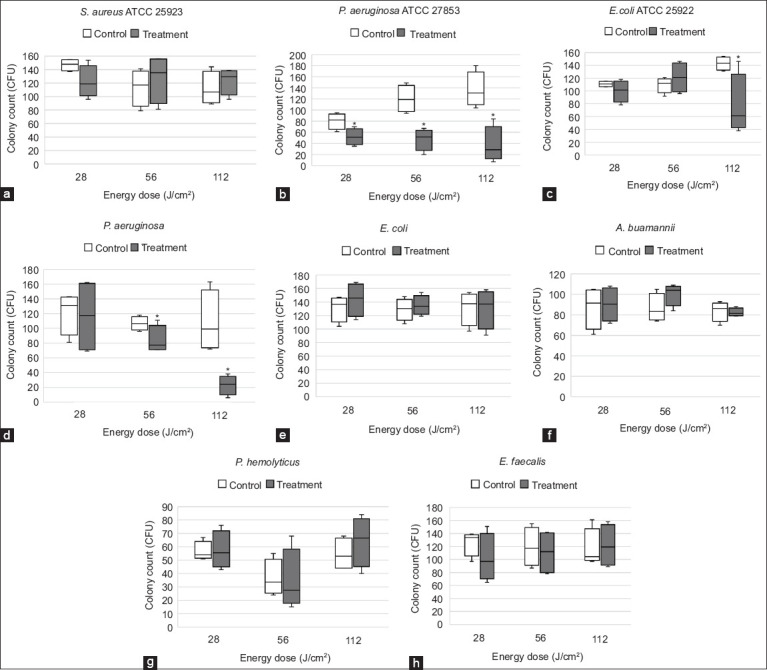
Box plots graph showing bacterial colony counts at 28, 56, and 112 J/cm^2^. The lines at each graph represent colony counts of individual plate (n = 4) for (a) *S. aureus* (ATCC 25923), (b) *P. aeruginosa* (ATCC 27853), (c) *E. coli* (ATCC 25922), (d) *P. aeruginosa*, (e) *E. coli*, (f) *A. baumannii*, (g) *S. haemolyticus*, and (h) *E. faecalis*. *There was a significant reduction in colony count between treatment (irradiated plate) and control (non-irradiated plate) in *P. aeruginosa* ATCC 27853 at all energy doses, *E. coli* (ATCC 25922) at 112 J/cm^2^, and *P. aeruginosa* at 56 and 112 J/cm^2^ (p < 0.05). *S. aureus*=*Staphylococcus aureus*, *E. coli=Escherichia coli, P. aeruginosa=Pseudomonas aeruginosa, A. baumannii=Acinetobacter baumannii, S. haemolyticus=Staphylococcus haemolyticus, E. faecalis=Enterococcus faecalis*, ATCC=American Type Culture Collection.

Bacterial isolates obtained from canine wounds in this study included *P. aeruginosa*, *Acinetobacter baumannii*, *Staphylococcus haemolyticus*, *E. coli*, and *Enterococcus faecalis*. The mean colony counts, SDs, and percentage reductions for both treated and control groups are summarized in Table 2. Significant reductions in bacterial growth were observed for *E. coli* (ATCC 25922) at an energy dose of 112 J/cm^2^, *P. aeruginosa* (ATCC 27853) at all tested energy doses, and clinical isolates of *P. aeruginosa* at 56 and 112 J/cm^2^ (p < 0.05) (Figure 1). In contrast, no statistically significant inhibition was detected for *S. aureus* (ATCC 25923), *A. baumannii*, *S. haemolyticus*, clinical *E. coli*, or *E. faecalis* at any of the energy levels applied.

**Table 2:** Average colony count (colony-forming units), SD, percentage reduction, and p*-*value between treatment and control groups for each energy dose.

Bacteria species	Control group (mean ± SD) (n = 4)	Treatment group (Mean ± SD) (n = 4)	% Reduction	p-value
*S. aureus* (ATCC 25923)				
28 J/cm^2^	147.0 ± 8.9	122.0 ± 24.0	−17.0	0.085
56 J/cm^2^	113.5 ± 27.2	127.0 ± 35.9	+11.8	0.574
112 J/cm^2^	111.8 ± 24.5	123.5 ± 19.8	+10.1	0.632
*E. coli* (ATCC 25922)				
28 J/cm^2^	110.8 ± 4.4	99.8 ± 16.9	−9.9	0.263
56 J/cm^2^	109.3 ± 12.3	121.0 ± 23.8	+10.8	0.458
112 J/cm^2^	142.8 ± 10.6	76.5 ± 47.8	−46.4	0.045
*P. aeruginosa* (ATCC 27853)				
28 J/cm^2^	80.0 ± 14.4	51.0 ± 14.7	−36.3	0.010
56 J/cm^2^	120.3 ± 24.2	47.5 ± 19.8	−60.5	0.018
112 J/cm^2^	136.5 ± 31.8	23.5 ± 32.9	−82.8	0.000
*A. baumannii*				
28 J/cm^2^	87.2 ± 20.5	90.3 ± 17.0	+3.4	0.366
56 J/cm^2^	86.5 ± 13.9	100.3 ± 11.1	+15.9	0.258
112 J/cm^2^	83.8 ± 9.8	82.5 ± 4.00	−1.5	0.817
*P. aeruginosa*				
28 J/cm^2^	121.5 ± 28.8	116.5 ± 50.6	−4.1	0.758
56 J/cm^2^	106.8 ± 9.2	84.3 ± 18.6	−21.1	0.026
112 J/cm^2^	108.3 ± 42.0	23.0 ± 13.22	−78.8	0.011
*S. haemolyticus*				
28 J/cm^2^	56.5 ± 7.2	57.5 ± 14.2	+2.2	0.900
56 J/cm^2^	36.5 ± 13.6	34.5 ± 23.1	−5.5	0.780
112 J/cm^2^	54.5 ± 12.4	64.3 ± 18.7	+18.3	0.199
*E. coli*				
28 J/cm^2^	131.0 ± 19.4	143.8 ± 24.0	+9.7	0.492
56 J/cm^2^	129.0 ± 16.5	135.0 ± 14.5	+4.7	0.475
112 J/cm^2^	131.5 ± 25.0	130.8 ± 29.4	−0.6	0.974
*E. faecalis*				
28 J/cm^2^	125.8 ± 19.5	102.5 ± 36.6	−18.5	0.218
56 J/cm^2^	119.3 ± 30.3	111.0 ± 33.7	−6.9	0.734
112 J/cm^2^	116.8 ± 29.7	121.5 ± 33.3	+4.1	0.790

*S. aureus=Staphylococcus aureus, E. coli=Escherichia coli, P. aeruginosa=Pseudomonas aeruginosa, A. baumannii=Acinetobacter baumannii,* *S. haemolyticus=Staphylococcus haemolyticus, E. faecalis=Enterococcus faecalis*, SD=Standard deviation, ATCC=American Type Culture Collection

## DISCUSSION

### Species- and dose-dependent bactericidal efficacy

Blue light demonstrated notable bacterial reduc-tion capabilities in this study. However, its effectiveness varied depending on the bacterial species, strain, and energy dose applied. Specifically, an energy dose of 112 J/cm^2^ resulted in 46.5% inhibition of *E. coli* (ATCC 25922), while *P. aeruginosa* (ATCC 27853) exhibited dose-dependent inhibition at 36.3%, 60.5%, and 82.8% for 28, 56, and 112 J/cm^2^, respectively. Clinical isolates of *P. aeruginosa* from canine wounds exhibited significant inhibition at 56 J/cm^2^ (21%) and 112 J/cm^2^ (78.7%) compared to the control groups. In contrast, no signi- ficant reduction was observed in *S. aureus* (ATCC 25923), *A. baumannii*, *S. haemolyticus*, clinical *E. coli*, or *E. faecalis* across any energy dose. These findings contrast with previous studies by Schnedeker *et al*. [24], which showed the higher efficacy of 465-nm blue light against MRSA and 450-nm light against *S. aureus* at much lower energy doses [26]. Similarly, Halstead *et al*. [27] and Lui *et al*. [28] have reported the successful inhibition of *A. baumannii* and *E. faecalis* at wavelengths between 400 and 455-nm. Nonetheless, our study confirms the selective efficacy of 465-nm blue light against *E. coli* (ATCC 25922) and *P. aeruginosa*, including clinical wound isolates.

### Role of bacterial photosensitizers

The antimicrobial mechanism of blue light pri- marily involves activation of endogenous photosensitizers, such as porphyrins, within bacterial cells [29]. These chromophores, once photoexcited, produce reactive oxygen species (ROS) that lead to cell death. The effectiveness of blue light depends on the amount of porphyrins produced, which varies among bacterial species. *P. aeruginosa* is known to generate higher porphyrin levels than *S. aureus* and *E. coli* [30], which aligns with the observed efficacy of blue light in our study. Strain variability also influences sensitivity, as those producing more chromophores and ROS are more susceptible to photoinactivation [31]. Our findings showed that even within the same species, clinical and standard strains responded differently, justifying our comparative evaluation. However, quantifying porphyrin levels was beyond the scope of this investigation.

### Gram staining and unexpected sensitivities

Contrary to the common assumption that Gram-negative bacteria are more resistant to blue light than Gram-positive bacteria [4, 21, 32], our study found significant inhibition of Gram-negative species (*P. aeruginosa* and *E. coli*) and no notable inhibition of the Gram-positive *S. aureus* and *E. faecalis*. This observation suggests that bacterial response to blue light cannot be generalized solely based on Gram’s classification.

### Influence of wavelength on efficacy

Wavelength plays a critical role in determining the bactericidal efficacy of blue light. Shorter wave-lengths (400–420 nm) have been shown to possess higher antimicrobial activity than longer ones (450–470 nm) [33–36]. This is attributed to the abso-rption characteristics of bacterial chromophores. For instance, at 420-nm, a 99% reduction in biofilm-forming bacteria was achieved, whereas at 455 and 480-nm, reductions were only 60%–83% [33]. Similarly, the effective inhibitory doses against *Saccharomyces cerevisiae* were 182 J/cm^2^ at 405-nm and 527 J/cm^2^ at 450-nm, with porphyrins and flavins acting as the dominant photosensitizers, respectively [34]. These differences are likely due to the higher photon energy of shorter wavelengths and their alignment with the Soret absorption band of porphyrins (peaking at 405–420 nm) [37, 38]. Therefore, the relatively lower efficacy of 465-nm blue light in this study may be a result of suboptimal chromophore activation compared to shorter wavelengths.

### Effect of power density and exposure duration

Besides wavelength, power density significantly affects the bactericidal potential of blue light. In this study, a relatively low power density of 31 mW/cm^2^ was used, necessitating 60 min to deliver 112 J/cm^2^ for effective inhibition of *P. aeruginosa* and *E. coli* (ATCC 25922). By contrast, De Sousa *et al*. [26] achieved inhibition of *S. aureus*, *P. aeruginosa*, and *E. coli* using 450-nm light at only 6 J/cm^2^ with a power density of 350 mW/cm^2^. Halstead *et al*. [27] also reported effective inhibition of *A. baumannii* using 400-nm light at 61 mW/cm^2^. A separate study by Leder *et al*. [19] demonstrated that the same energy dose (144 J/cm^2^) delivered at a higher power density (40 mW/cm^2^) was more effective in treating *P. aeruginosa* in human wounds than when delivered at a lower intensity over a longer period. In addition, research on *Porphyromonas gingivalis* has confirmed that higher power density enhances bactericidal outcomes, even when total energy remains constant [20]. These findings highlight the potential benefits of using high-power-density devices for more efficient clinical applications.

### Limitations

The adaptive capability of bacteria to external stressors [39], coupled with variables such as wound debris, hair, and host immune responses, may reduce the clinical effectiveness of blue light observed *in vitro*. Moreover, the complexity of factors influencing efficacy, including energy dose, wavelength, power density, and bacterial variability, makes it challenging to establish universal treatment protocols.

This study’s limitations include the use of a low-power-density blue light device, resulting in prolonged exposure times that may not be ideal for clinical settings. Additionally, sample collection was not controlled for prior antibiotic exposure, which could affect bacterial susceptibility. Despite these limitations, blue light re- mains a promising adjunctive therapy. Identifying the specific bacterial pathogen before treatment could enable tailoring of the energy dose for maximum effect. Future research should expand to include a broader range of bacterial pathogens and evaluate the efficacy of high-power blue light devices under clinical conditions.

## CONCLUSION

This study demonstrated that 465-nm blue light possesses selective bactericidal efficacy, parti-cularly against *P. aeruginosa* (ATCC 27853 and clinical isolates) and *E. coli* (ATCC 25922). Significant bacterial inhibition was observed at higher energy doses, with *P. aeruginosa* showing a reduction of up to 82.8% and *E. coli* (ATCC 25922) achieving a 46.4% reduction at 112 J/cm^2^. Clinical isolates of *P. aeruginosa* also showed significant inhibition at 56 and 112 J/cm^2^. In contrast, *S. aureus*, *A. baumannii*, *S. haemolyticus*, clinical *E. coli*, and *E. faecalis* exhibited no significant reduction across tested doses, highlighting species- and strain-dependent variability in response.

From a practical standpoint, 465-nm blue light offers a non-invasive, drug-free modality that could be integrated into veterinary wound management protocols, especially in cases involving *P. aeruginosa* or *E. coli*. It may serve as an adjunctive or alternative therapy to antibiotics, particularly valuable in settings where antimicrobial resistance is prevalent.

A major strength of this study lies in its compa-rative analysis of standard bacterial strains and clinical wound isolates under controlled, reproducible conditions. In addition, the use of quantified energy doses and standardized methodology enhances the relevance of findings for translational and clinical applications.

Future research should focus on the development and evaluation of high-power blue light devices with shorter wavelengths and reduced exposure time for better clinical feasibility. Further *in vivo* studies are needed to confirm efficacy in real-world wound environments and to optimize treatment protocols tailored to specific pathogens.

In summary, 465-nm blue light exhibits promising bactericidal potential against select pathogens, suppo-rting its role as a viable adjunct to conventional antimi-crobial strategies in veterinary practice. Broader validation and refinement of this approach may pave the way for more effective and targeted phototherapy applications in both veterinary and human medicine.

## Data Availability

All the generated data are included in the manuscript.

## AUTHORS’ CONTRIBUTIONS

NK, SH, SS, DKB, SC, SJ, TS, DK, and PN: Conceptualized and designed the study. PN, DKB, and SC: Performed the study. SS: Statistical analysis. PN and SS: Analyzed and interpreted data. DK, NK, SH, SS, PJ, SJ, TS, CN, NB, PW, DK: Supervised this study. DK: Project administration. PN, NK, and SH: Drafted the manuscript. PN, DK, NK, SH, and TS: Critically revised the manuscript. All authors have read and approved the final manuscript.
